# Assessing Variances in Dentist's Interpretations of Antibiotic Prophylaxis Guidelines: A Survey of Dental School Faculty

**DOI:** 10.1155/ijod/9355907

**Published:** 2025-01-29

**Authors:** Jennifer Bereckis, Susan Rowan, Danny Hanna, Aniruddh Narvekar, Anne Koerber

**Affiliations:** ^1^Department of Restorative Dentistry, College of Dentistry, University of Illinois, Chicago, Illinois, USA; ^2^Department of Oral Medicine and Diagnostic Sciences, College of Dentistry, University of Illinois, Chicago, Illinois, USA; ^3^Department of Periodontics, College of Dentistry, University of Illinois, Chicago, Illinois, USA

**Keywords:** antibiotic prophylaxis, dentistry, gingival manipulation, infective endocarditis

## Abstract

**Purpose:** A recent review by the American Heart Association (AHA) noted a decrease in the prescription of antibiotic prophylaxis (AP) for infective endocarditis (IE) following the release of their guidelines in 2007. However, studies indicate dentists may still face challenges in identifying which procedures require AP and which procedures are considered to involve gingival manipulation (GM) requiring the need for AP.

**Methods:** A sample of dental school faculty was surveyed to assess their likelihood of prescribing AP for various dental procedures when treating individuals at high risk for IE and their perception of the likelihood of those procedures involving GM.

**Results:** A total of 134 individuals responded to the survey. Consensus on AP was not achieved for eight of 24 procedures, and consensus on GM was not achieved for four out of 24 procedures.

**Conclusions:** Data gathered revealed a difference of opinions among dental faculty concerning the appropriate dental procedures warranting the prescription of AP for patients at risk of IE. Similarly, there was a lack of consensus among dental faculty regarding dental procedures specifically involving GM. The discordance observed between these two categories implies a potential lack of clarity in the 2007 AHA guidelines. The criteria related to GM for determining the necessity of AP in high-risk individuals may introduce confusion for dental faculty, possibly extending to dentists practicing in nonacademic settings. Such ambiguity can potentially contribute to inappropriate decision-making regarding the prescription of AP.

## 1. Introduction

Dental procedures have the potential to contribute to the development of infective endocarditis (IE) due to bacteremia following dental procedures and an elevated recovery of viridans streptococci in bacterial cultures found in individuals diagnosed with IE [1–3]. Reports of links between IE and dental procedures date back as far as a century ago [4]. In fact, a few studies [5, 6] have found that “following a dental extraction, 61% of patients had a positive blood culture for oral viridans group Streptococci.” This discovery influenced the American Heart Association's (AHA) first recommendation for antibiotic prophylaxis (AP) to prevent IE. This occurred in 1955 when the AHA published guidelines recommending the prescription of antibiotics prophylactically for patients undergoing dental procedures with a high risk of developing IE [7]. Over the next two decades, there were many updates to the guidelines, modifying the type of antibiotic recommended, number of doses and route of administration [4, 8]. In 1972, for the first time, the American Dental Association (ADA) endorsed the published AHA guidelines [9]. There were four more reiterations of the guidelines between 1972 and 1997 [7–15]. In August 2007, with recommendations made by the ADA, the AHA updated the guidelines for IE prophylaxis related to dental procedures. These guidelines were updated by a scientific statement in 2021 by the AHA that recommended no changes to the 2007 guideline recommendations [16]. The 1997 guideline detailed which dental procedures were likely to cause a bacteremia [17]. In contrast, the current guideline relies on the provider to distinguish whether dental procedures involve manipulation of gingival tissue, the periapical region of teeth, or perforation of the oral mucosa [16].

The current guidelines were driven by the significant rise in antibiotic-resistant strains of viridans group streptococci with the goal of restricting the prescription of antibiotics to only situations where they are deemed essential. The number of antibiotic-resistant infections acquired annually is considerable and is associated with significant mortality [17]. Since dentists prescribe at least 10% of all antibiotic prescriptions [18], it is important that dentists, along with other health professionals, adhere to strict prescription guidelines. The risks of adverse outcomes of IE from a procedure must be weighed with the risks of adverse outcomes from antibiotic usage to determine whether antibiotics should be prescribed. A review of the 2007 guidelines found that dentists decreased their rate of prescribing for IE following the publication of the guidelines [19].

However, the decision of dentists to prescribe depends not only on identifying high-risk patients but also on their ability to identify those dental procedures that bring a risk of IE. The 2007 guidelines state that AP is appropriate for a high-risk patient for “all dental procedures that involve manipulation of gingival tissue or the periapical region of teeth or perforation of the oral mucosa.” One of the major concerns of these guidelines is that they allow for varying interpretations among dentists, leading to less-than-optimal care and patient safety. The guidelines exclude AP for “routine anesthetic injections through noninfected tissue, taking dental radiographs, placement of removable prosthodontic or orthodontic appliances, placement of orthodontic brackets shedding of primary teeth, and bleeding from trauma to the lips or oral mucosa.” A previous study found that dentists and hygienists do not agree about which procedures would call for AP [20]. The criteria appear to specify the need for AP in surgical procedures for appropriate patients. However, the guidance for other dental procedures is less specific for procedures requiring gingival manipulation (GM). Therefore, this study explored whether dental faculty agreed regarding which procedures involve GM, which procedures would call for AP, and whether the two criteria were congruent.

## 2. Methods and Materials

A survey was conducted using a convenience sample of dental faculty from accredited dental schools within the United States and Canada. An IRB exemption was granted by the University of Illinois Chicago, Office for the Protection of Research Subjects (# 2019-0756). The survey was distributed electronically through the American Dental Education Association (ADEA) Clinical Administrative listserv, requesting dental school administrators to share the link with dental faculty. The survey was developed using Qualtrics software (Provo, UT, USA) and administered via email. The survey link remained active for a duration of 4 weeks, during which two reminders were sent. All respondents remained anonymous. This method did not allow for the determination of the response rate since we could not determine how many faculty members received the questionnaire.

Following the initial determination of eligibility for the study of the respondents, the questionnaire began, “Given a patient who qualifies for antibiotic prophylaxis,” and then asked two categories of questions. The first category asked the subject to rate the likelihood of prescribing AP for a list of routine dental procedures. The second category asked about the likelihood of the same procedures involving GM. We did not include procedures such as periodontal surgery or extraction, for which the guidelines are more clearly stated.

Responses were collapsed into binary responses, with “*50/50 Likelihood*,” “*Moderate Likelihood*,” and “*Very Likely*” collapsed into “*yes, would prescribe*,” or “*yes would involve GM*,” “*Not likely*,” and “*Some Likelihood*” were collapsed into “*no, would not prescribe*,” or “*no, would not involve GM*.” We chose 75% as the criterion to establish consensus among faculty for whether AP would be prescribed or whether GM would be involved.

### 2.1. Data Analysis

The survey data were compiled and recorded on Qualtrics. Consensus was reached when 75% of respondents agreed regarding the prescription of AP. Data were also analyzed similarly to determine the likelihood that a procedure would produce GM, and consensus was reached when 75% of respondents agreed. Once the percentages were obtained, a Wilson Score Interval test was determined at a 95% confidence interval (CI). This was done using the Statistics Kingdom proportion CI calculator website tool.

## 3. Results

The survey questionnaire was distributed to 78 administrators of accredited dental schools within the United States and Canada. While the exact number of faculty who received the questionnaire remains unknown, the study garnered 134 responses. However, four of the received questionnaires were incomplete, leaving 130 responses for analysis.

Table 1 displays the demographic information of the subjects. Most respondents were dentists, with 10% representing dental hygienists and others. Among the respondents, half reported general dentistry as their scope of practice, and two-thirds were full-time faculty. In addition, 91% reported that they prescribe or recommend antibiotics. There was a higher representation of male respondents compared to female respondents. Most of the respondents reported that they worked in the clinic three to four days a week. Approximately half of the subjects had graduated 30 years or more ago, indicating that most of them completed their education before the 1997 guidelines were implemented. Additionally, about 22% of the respondents had graduated after the 2007 guidelines were implemented.

Table 2 lists the dental procedures in the survey, ranked by the percentage of faculty likely to prescribe antibiotics for that procedure in an eligible patient. Using the definition of consensus as 75%, 16 procedures out of 24 produced a consensus for AP.

The next set of questions asked the likelihood of a procedure involving GM, listed in the questionnaire in the same order as the questions on AP.  Table 3 displays the procedures ranked by percent agreement on the involvement of GM. Consensus was reached for 20 of the 24 procedures.  Table 4 lists the nine procedures on which consensus was achieved both for GM and for prescribing AP, as well as the seven procedures on which a consensus was achieved for not involving GM and not prescribing AP.  Table 5 lists the four procedures for which faculty agreed to involve GM but did not agree on whether AP was necessary.  Table 6 lists the four procedures upon which faculty could not agree either on GM or the need for prophylactic antibiotics.

## 4. Discussion

This study [21] sought to assess the consensus among dental faculty on interpreting the 2007 AHA guidelines regarding prescribing prophylactic antibiotics for individuals with specific medical conditions planning to undergo dental procedures. The focus was determining agreement among faculty members on procedures involving GM and identifying which procedures necessitated AP for high-risk patients. For this survey, we focused solely on GM as a criterion, assuming that there would be agreement among dental faculty on more invasive procedures requiring AP. The survey revealed the heterogenicity of judgment for eight procedures regarding the need for AP and the heterogenicity of judgment for four procedures involving GM. Interestingly, there were four procedures that faculty assessed as involving GM but not requiring AP. All four of those procedures are either excluded by the 2007 or 1997 guidelines as requiring AP. These findings suggest that the criterion of GM could be confusing as a criterion for AP.

For the procedures that dental faculty were not in agreement, either on the need for AP or on the involvement of GM; it is possible that these procedures require more assessment on a case-by-case basis to determine the need for AP. The findings from our study are similar to those reported in a previous study [20].

Interestingly, while there is more agreement about procedures that produce GM, there is still a significant lack of agreement on when to prescribe AP. Root canal therapy barely achieved the status of consensus for antibiotic treatment. This is notable because the 1997 guidelines specifically recommend AP for root canal instrumentation beyond the apex. The 2007 guidelines recommend AP for dental procedures involving the apexes of teeth. In essence, both guidelines recommend AP for root canal procedures, yet there is barely a consensus among faculty regarding AP for those procedures. The same can be said about routine prophylaxis (dental cleaning). There was 84% agreement that routine prophylaxis involved GM, but the likelihood of prescribing AP barely made consensus. This is remarkable because the 1997 guidelines explicitly recommend AP for routine prophylaxis; however, the 2007 guidelines do not mention routine prophylaxis. Other noteworthy procedures that did not achieve agreement to produce GM nor prescribe AP were restorative procedures involving an implant, placement of orthodontic bands, crown delivery, and treatment of cervical caries 1 mm above the gingival margin. Again, the 1997 guidelines specifically state AP is recommended for the initial placement of orthodontic bands. The 2007 update does not mention orthodontic bands; however, it states that AP is not recommended for the placement of orthodontic brackets. The guidelines have left prescribing for AP unclear for dentists. Although treatment of proximal caries on a posterior tooth, a procedure involving placement of rubber dam, placement of stainless-steel crown on the primary tooth, and treatment of proximal caries on an anterior tooth all achieved consensus to produce GM, a consensus for AP was not reached. The 2007 guidelines recommend AP for procedures that involve GM; therefore, all four procedures should have reached a consensus for AP prescribing. The 1997 guidelines specifically list restorative dentistry (operative and prosthodontic) with or without a retraction cord as a procedure for AP is not recommended. A plausible explanation for the variance in response could be that dentists still refer to the 1997 guidelines. These findings are consistent with previous studies that have found inconsistency and controversy regarding when to prescribe AP [22]. Similar to the AHA guidelines, the European Society of Cardiology recommends AP for “manipulation of the gingival or periapical region of the teeth or perforation of the oral mucosa,” while the National Institute for Health and Care Excellence considers only “extraction and deep descaling” as high-risk procedures for AP [23, 24]. According to Jain et al. [20], many dentists disagree with current guidelines and feel they are too vague. In one study, dentists defined invasive procedures to include extractions, periodontal procedures, implant placement, and dental cleanings [25]. This study concluded that routine prophylaxis (dental cleaning) does not produce GM yet there was near consensus to prescribe AP. Additionally, a previous study has shown that 70% of respondents reported still prescribing AP even though the 2007 guidelines no longer indicated it [22].

Clinical compliance as it relates to prescribing AP is unclear [22]. Some dentists and hygienists admit to prescribing AP even though it is not recommended due to patient or caregiver pressure [26]. The pressure to write the prescription may often come from the physician or cardiologist, even though the cardiologist lacks the necessary understanding of dental procedures to make that recommendation [20, 22, 25]. Dentists also reported that nonclinical factors, such as precautionary measures for patients on vacation, legal concerns, and patient demands, contributed to noncompliance with recommended guidelines [25]. While there is no mention of bleeding in the 2007 guidelines, one study states that dentists prescribe AP when bleeding is anticipated [22]. Random exposures to bacteremia from daily activities are more likely to cause IE than those related to dental procedures [25]. One source suggests that regular tooth brushing creates a greater risk of IE than a single dental procedure due to repetitive bacteremia [26]. Adverse effects of antibiotics and the possibility of antibiotic resistance were not reported as influencing prescribing decisions [25].

Some of the limitations of our study should be acknowledged. Our target population included a convenience sample of dental school faculty, and therefore, the sample size was small and may not be representative of all dental school faculty and dental providers. Additionally, we could not calculate a response rate nor determine if the questionnaire was distributed to all dental faculty in all the schools. Despite these limitations, this study offers insights indicating a lack of consensus among dental faculty regarding procedures involving GM and the necessity for AP for these procedures. A major concern is that if such confusion occurs among dental faculty, there is potentially greater confusion among dentists in general.

Effectively addressing these issues necessitates a comprehensive approach. This involves implementing enhanced communication and training initiatives to improve the understanding of current AP guidelines, seeking expert consultation from specialists in the field, establishing decision-making protocols, and fostering collaborative discussions through open forums. Adopting these strategies has the potential to cultivate a more collaborative environment, ultimately leading to increased adherence to the latest best practices in AP for patients at high risk for the development of IE.

## 5. Conclusions

This study on a convenience sample of dental school faculty found a lack of consensus regarding which dental procedures justify the prescription of AP. While fewer procedure types lacked consensus for GM, both categories of questions should have provided similar results. The lack of concordance between the two categories suggests that the 2007 guidelines are unclear for dental faculty members in determining the need for AP. To address these challenges, the authors recommend a multifaceted strategy involving enhanced communication, expert consultation, and ongoing training for aligning practices with evolving standards.

## Figures and Tables

**Table 1:** Professional and demographic data of respondents.

Profession	*n* (%)
Dentists	109 (89)
Hygienists	9 (7)
Other	4 (3)
Total (8 missing)	122 (100)

Specialty or Scope	*n* (%)

General dentistry	62 (51)
Prosthodontics	14 (11)
Endodontics	9 (7)
Pediatrics	4 (3)
Orthodontics	3 (2)
Oral medicine	2 (2)
Oral surgery	2 (2)
Public health	2 (2)
Other	24 (20)
Total (8 missing)	122 (100)

Years since professional school graduation	*n* (%)

0–5 years	8 (7)
6–10 years	10 (8)
11–15 years	8 (7)
16–20 years	13 (11)
21–25 years	8 (7)
26–30 years	12 (10)
<30 years	62 (51)
Total (9 missing)	121 (101)

Sex	*n* (%)

Male	69 (57)
Female	53 (43)
Total (8 missing)	122 (100)

Faculty status	*n* (%)

Full time	80 (66)
Part time	42 (34)
Total (8 missing)	122 (100)

Do you ever recommend or prescribe antibiotics?	*n* (%)

Yes	116 (91)
No	11 (9)
Total (3 missing)	127 (100)

Days/week providing or supervising clinical dentistry	*n* (%)

None	11 (9)
1–2 days	38 (31)
3–4 days	51 (42)
5+ days	22 (18)
Total (8 missing)	122 (100)

**Table 2:** Percentage of respondents likely to prescribe antibiotic prophylaxis for an eligible patient by procedure type.

Procedure	Percent likely to prescribe (95% CI)
Implant placement	91 (85%–95%)
Extraction of permanent teeth	91 (85%–95%)
Scaling and root planning	90 (84%–94%)
Extraction of primary teeth	85 (78%–90%)
Treatment of cervical caries extending below the gingival margin	79 (71%–85%)
Crown preparation	76 (68%–83%)
Oral exam with probing	75 (68%–81%)
Routine prophylaxis	74 (66%–81%)
Root canal therapy	70 (62%–77%)
Treatment of proximal caries on a posterior tooth	63 (55%–71%)
Placement of stainless-steel crown on primary tooth	63 (57%–73%)
Procedure involving placement of rubber dam	62 (54%–70%)
Restorative procedure involving an implant	57 (49%–66%)
Treatment of proximal caries on an anterior tooth	56 (47%–64%)
Treatment of cervical caries 1 mm above the gingival margin	45 (37%–54%)
Placement of orthodontic bands	40 (31%–48%)
Crown delivery	39 (31%–47%)
Treatment of caries on a buccal pit	28 (21%–36%)
Treatment of caries on an occlusal surface	25 (17%–31%)
Taking oral impressions	18 (12%–25%)
Oral exam without probing	4 (2%–9%)
Taking 1 PA radiograph	4 (2%–10%)
Fluoride treatment	3 (1%–8%)
Taking 1 panoramic radiograph	3 (1%–8%)

Abbreviation: CI, confidence interval.

**Table 3:** Percentage of various dental procedures involving gingival manipulation as assessed by respondents.

Procedure	Percent likely to involve gingival manipulation (95% CI)
Implant placement	98 (94%–100%)
Extraction of permanent teeth	97 (93%–99%)
Scaling and root planning	95 (89%–98%)
Extraction of primary teeth	89 (91%–99%)
Treatment of cervical caries extending below the gingival margin	89 (82%–93%)
Crown preparation	86 (87%–91%)
Routine prophylaxis	84 (76%–90%)
Oral exam with probing	83 (75%–88%)
Treatment of proximal caries on a posterior tooth	83 (65%–80%)
Procedure involving placement of rubber dam	75 (67%–82%)
Placement of stainless-steel crown on primary tooth	75 (67%–82%)
Treatment of proximal caries on an anterior tooth	67 (58%–75%)
Root canal therapy	67 (58%–75%)
Restorative procedure involving an implant	66 (57%–74%)
Placement of orthodontic bands	54 (45%–63%)
Crown delivery	50 (41%–59%)
Treatment of cervical caries 1 mm above the gingival margin	49 (40%–58%)
Taking oral impressions	28 (20%–37%)
Treatment of caries on an occlusal surface	16 (10%–23%)
Treatment of caries on a buccal pit	16 (10%–24%)
Oral exam without probing	4 (2%–10%)
Taking one periapical radiograph	4 (2%–10%)
Fluoride treatment	3 (0%–7%)
Taking one panoramic radiograph	2 (0%–6%)

Abbreviation: CI, confidence interval.

**Table 4:** Dental procedures likely or unlikely to produce gingival manipulation and require the prescription of antibiotic prophylaxis as assessed by respondents.

Dental procedures assessed as likely to produce gingival manipulation and likely to prescribe antibiotic prophylaxis
1. Implant placement 2. Extraction of permanent teeth 3. Scaling and root planning 4. Extraction of primary teeth 5. Treatment of cervical caries extending below the gingival margin 6. Crown preparation 7. Routine prophylaxis 8. Oral exam with probing 9. Root canal therapy

Dental procedures assessed as not likely to produce gingival manipulation and not likely to prescribe antibiotic prophylaxis

1. Taking oral impressions 2. Treatment of caries on an occlusal surface 3. Treatment of caries on a buccal pit 4. Oral exam without probing 5. Taking one periapical radiograph 6. Fluoride treatment 7. Taking one panoramic radiograph

**Table 5:** Dental procedures likely to produce gingival manipulation but disagreement on prescription of antibiotic prophylaxis as assessed by respondents.

1. Treatment of proximal caries on a posterior tooth 2. Procedure involving placement of rubber dam 3. Placement of stainless-steel crown on primary tooth 4. Treatment of proximal caries on an anterior tooth

**Table 6:** Dental procedures that did not reach an agreement on the involvement of gingival manipulation or on prescription of antibiotic prophylaxis as assessed by respondents.

1. Restorative procedure involving an implant 2. Placement of orthodontic bands 3. Crown delivery 4. Treatment of cervical caries 1 mm above the gingival margin

## Data Availability

The datasets generated and/or analyzed during the current study are available from the corresponding author upon reasonable request.
